# Pathology of the carotid space

**DOI:** 10.1186/s13244-019-0704-z

**Published:** 2019-02-15

**Authors:** Harris U. Chengazi, Alok A. Bhatt

**Affiliations:** Department of Imaging Sciences, University of Rochester Medical Center, 601 Elmwood Avenue, P.O. Box 648, Rochester, NY 14642 USA

**Keywords:** Carotid space, Paraganglioma, Meningioma, Carotidynia

## Abstract

The complex anatomy of the carotid space within a small confined area is unique to the head and neck and allows for a vast array of pathology. This paper will review the anatomy of the carotid space from the skull base to the thorax, defining its borders at multiple levels, as well as its contents. The paper will also describe the myriad of mass lesions and vascular pathologies that may occur within the carotid space. The discussion will include anatomic considerations in differential diagnoses, imaging features, and lesion characteristics across multiple imaging modalities including CT, MRI, ultrasound, and conventional angiography. Entities discussed include paragangliomas, nerve sheath tumors, meningioma, fibromuscular dysplasia, carotidynia, thrombus, dissection, pseudoaneurysm, and pathology of the deep cervical chain lymph nodes. Understanding the complex and unique anatomy of the carotid space, as well as the nuances of navigating a broad differential, will empower the reader to make an accurate diagnosis.

## Key points

- A lesion centered within the carotid space will displace the ipsilateral parapharyngeal fat anteromedially.
- The infrahyoid carotid space contains the vagus nerve, as well as the common carotid artery and internal jugular vein.
- Carotid body tumors will splay the internal and external carotid arteries.
- Acute internal jugular vein thrombus can be associated with fluid in the retropharyngeal space.
- A metastatic cystic deep cervical chain lymph node may be from squamous cell carcinoma or papillary thyroid carcinoma.

## Introduction

The complex anatomy of the carotid space within a small confined space in the neck allows for a vast array of pathology. This article will review the anatomy of the carotid space, as well as the various types of pathology that may occur within this confined region. Masses within this space include paragangliomas, nerve sheath tumors, lipomas, and pathology involving the deep cervical chain lymph nodes. As the name implies, the carotid artery and jugular vein may also be involved as part of the pathology.

### Carotid space anatomy

The carotid space is a paired space defined by the carotid sheath, a connective tissue boundary in the neck, that is made by the superficial, middle, and deep layers of the deep cervical fascia. Extending from the jugular foramen at the skull base to the aortic arch at the thoracic inlet, the carotid space is divided craniocaudally into the supra- and infrahyoid regions. The suprahyoid carotid space is surrounded anteriorly by the masticator and parapharyngeal spaces, laterally by the parotid space, medially by the retropharyngeal space, and posteriorly by the perivertebral space. A mass centered within the carotid space will displace the parapharyngeal fat/space anteromedially [1].

The suprahyoid portion of the carotid space contains the internal carotid artery, the internal jugular vein, cranial nerves 9 through 12, the ansa cervicalis, the sympathetic plexus, and deep cervical lymph nodes [1]. The infrahyoid carotid space is surrounded anteriorly by the anterior cervical space, medially by the visceral and retropharyngeal spaces, and posteriorly by the perivertebral and posterior cervical spaces. Below the level of the hyoid, the ansa cervicalis (a loop of the first 3 cervical nerves) and cranial nerves 9, 11, and 12 have exited, thus leaving only cranial nerve 10. The internal jugular vein and the common carotid artery are also contained within the infrahyoid carotid space [2].

**Fig. 1:**
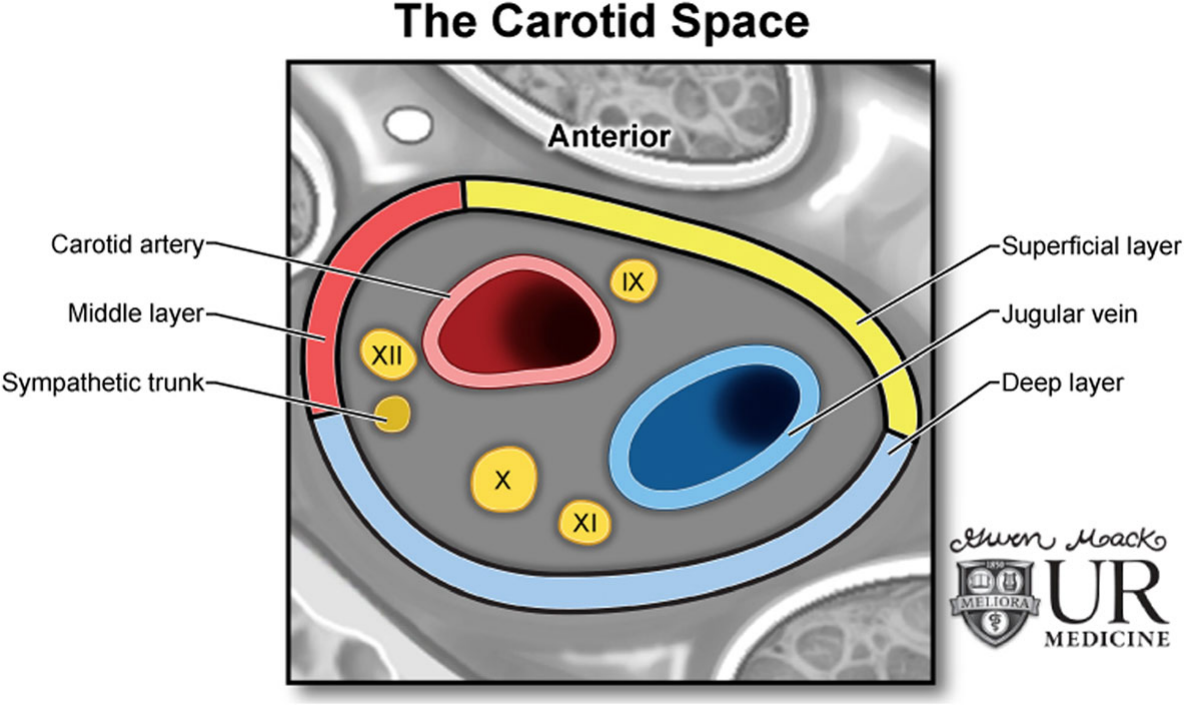
The carotid space. Illustration demonstrating the contents and configuration of the left carotid space, including cranial nerves IX, X, XI, and XII

Knowledge of the location of particular structures within the carotid space can lead to the correct diagnosis, if not narrow, the differential to a few lesions. The carotid artery is the center of the carotid space, and the jugular vein lies posterolateral to the carotid artery. The 10th cranial nerve lies in the posterior groove between these two vessels. The remaining cranial nerves 9, 11, and 12 all pierce the carotid sheath anteriorly. The ansa cervicalis is embedded in the anterior carotid sheath, and the sympathetic plexus is found posteriorly [3]. Lesions of the carotid space may arise from any of the above structures, and radiographic imaging is valuable in aiding diagnosis (Fig. 1).

### Paragangliomas

Paragangliomas, often referred to as glomus tumors, are rare neuroendocrine tumors that may occur anywhere in the body where healthy paraganglia occur. They are named for their location within the carotid sheath. These tumors tend to be well-marginated and highly vascular masses that are rubbery and firm on exam [4]. Paragangliomas can vary greatly in size and may be associated with germline mutations in the succinate dehydrogenase gene family; at least 30% of patients with a paraganglioma and no other risk factors may have a genetic mutation that increases the risk for these tumors [5, 6]. The majority of paragangliomas are nonfunctional. While functional tumors are rare, they can be life-threatening and may present clinically with signs of catecholamine hypersecretion [7]. Nonfunctioning tumors grow insidiously and present as palpable masses or pain at the site of the tumor [8]. It is important to remember that 10% of tumors may be clinically silent and are often detected incidentally on imaging studies [9]. Due to the highly vascular nature of these masses, biopsy carries significant risk; thus, their diagnosis on imaging is the key [10].

Due to their hypervascularity, paragangliomas typically demonstrate arterial spectral waveforms on ultrasound imaging, and intense homogenous contrast enhancement on computed tomography (CT) and magnetic resonance imaging (MRI). Feeding and draining vessels can also be identified on angiography. Larger tumors may enhance heterogeneously due to areas of necrosis and/or hemorrhage [6]. The classically described “salt and pepper” appearance of these tumors on MRI refers to high signal areas with slow flow or hemorrhage and low signal areas due to signal voids of tumor vessels classically described on T1-weighted imaging, but can also be on T2-weighted images [6].

#### Carotid body tumor

**Fig. 2:**
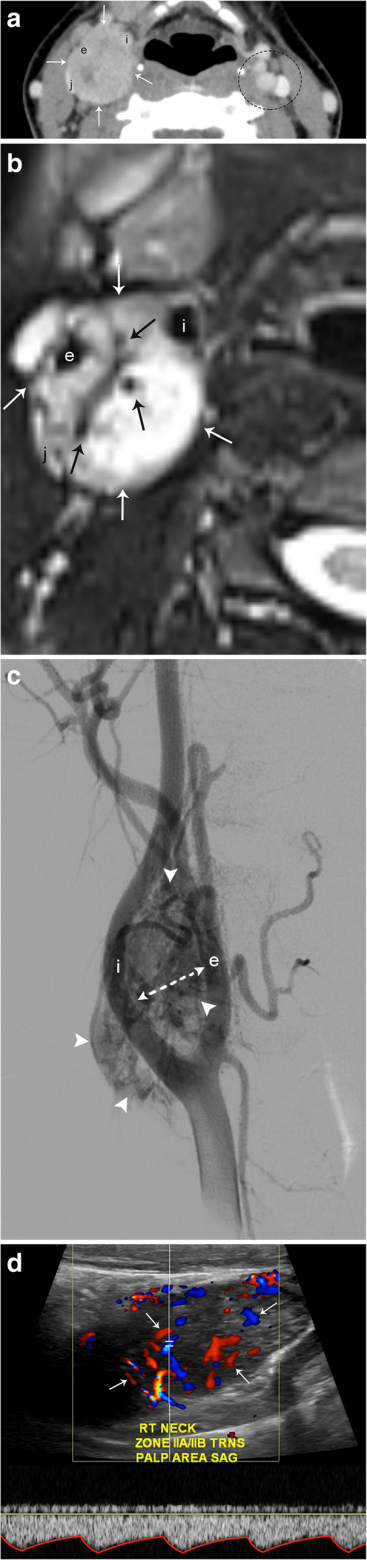
Carotid body tumor. **a** Axial CT image demonstrates a well-circumscribed, enhancing mass centered in the right carotid space (arrows) that causes splaying of the right internal (i) and external (e) carotid arteries. There is mass effect on the internal jugular vein (j). The left carotid space (dotted circle) demonstrates the normal relationship of the carotid and jugular vessels. **b** Axial T2-weighted MR image shows a well-circumscribed hyperintense mass (white arrows) with multiple internal flow voids (black arrows). **c** DSA demonstrates an intensely hypervascular mass (arrowheads) centered at the carotid bifurcation. Again seen is splaying of the internal and external carotid arteries. **d** Spectral Doppler ultrasound confirms hypervascularity (arrows) and internal arterial flow (spectral tracing) of this lesion

The carotid body is the largest collection of paraganglia in the head and neck and is found on the medial aspect of the carotid bifurcation bilaterally. Carotid body tumors are usually found in the fourth or fifth decade of life and are the most common head and neck paraganglioma. These tumors tend to grow slowly and painlessly and present as a lateral neck mass at the level of the carotid bifurcation. Given the lesion’s close proximity to cranial nerves 10–12, patients may also present with dysphagia, odynophagia, hoarseness of voice, or other cranial nerve deficits [11]. Due to their location, and when large enough, they tend to splay the internal and external carotid arteries apart from each other [4] (Fig. 2).

#### Glomus jugulare

**Fig. 3:**
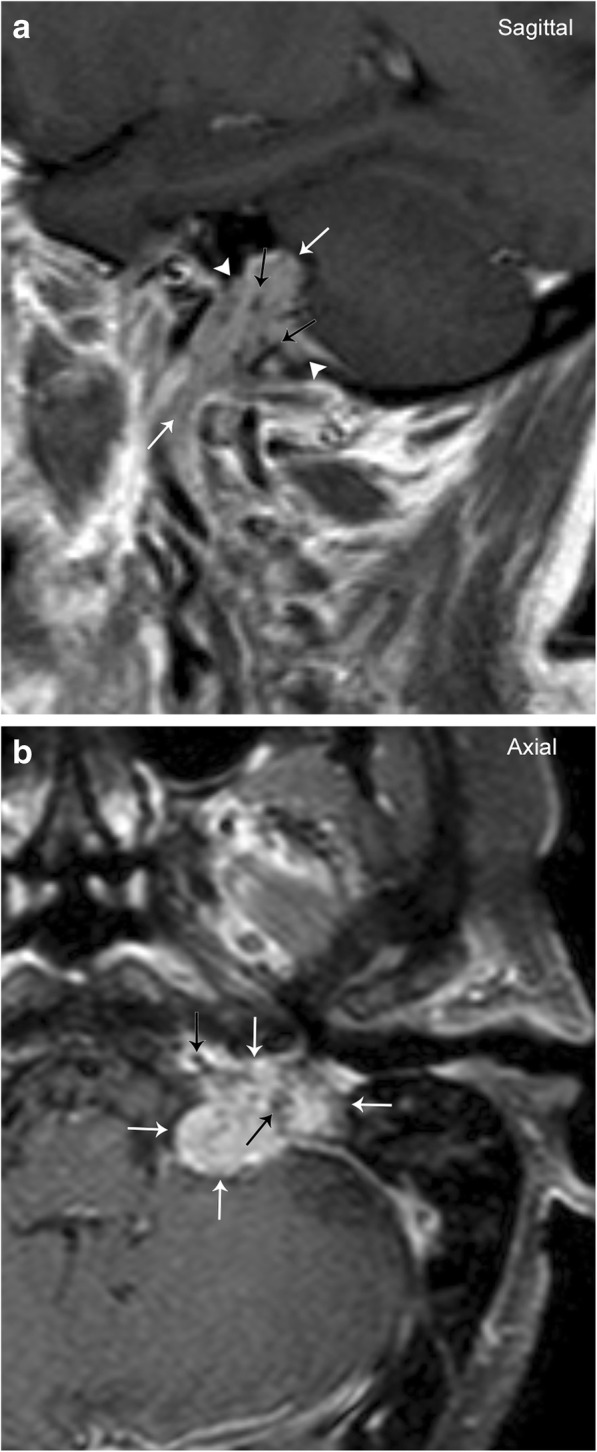
Glomus Jugulare. Sagittal (**a**) and axial (**b**) T1 post-contrast images demonstrate an enhancing mass (white arrows) centered at the jugular foramen (arrowheads). Note the prominent flow voids (black arrows) typical of a glomus tumor (paraganglioma)

Paragangliomas at the jugular foramen may arise from the tympanic branch of the glossopharyngeal nerve (Jacobson’s nerve), or the auricular branch of the vagus nerve (Arnold’s nerve), and are referred to as glomus jugulare (Fig. 3). These tumors have a 3:1 predilection for females over males and typically present in the fifth or sixth decades of life. Patients clinically present with pulsatile tinnitus, hearing loss or vertigo, and other symptoms related to the cranial nerves within the jugular foramen (glossopharyngeal, vagus, and accessory) [4]. Demineralization of the bony cortex surrounding the jugular foramen causes an enlargement of the foramen due to permeative, “moth-eaten” appearance of erosion on CT [6]. These tumors can extend into the tympanic cavity (glomus jugulotympanicum) and may also involve the internal carotid artery or internal jugular vein.

#### Glomus vagale

**Fig. 4:**
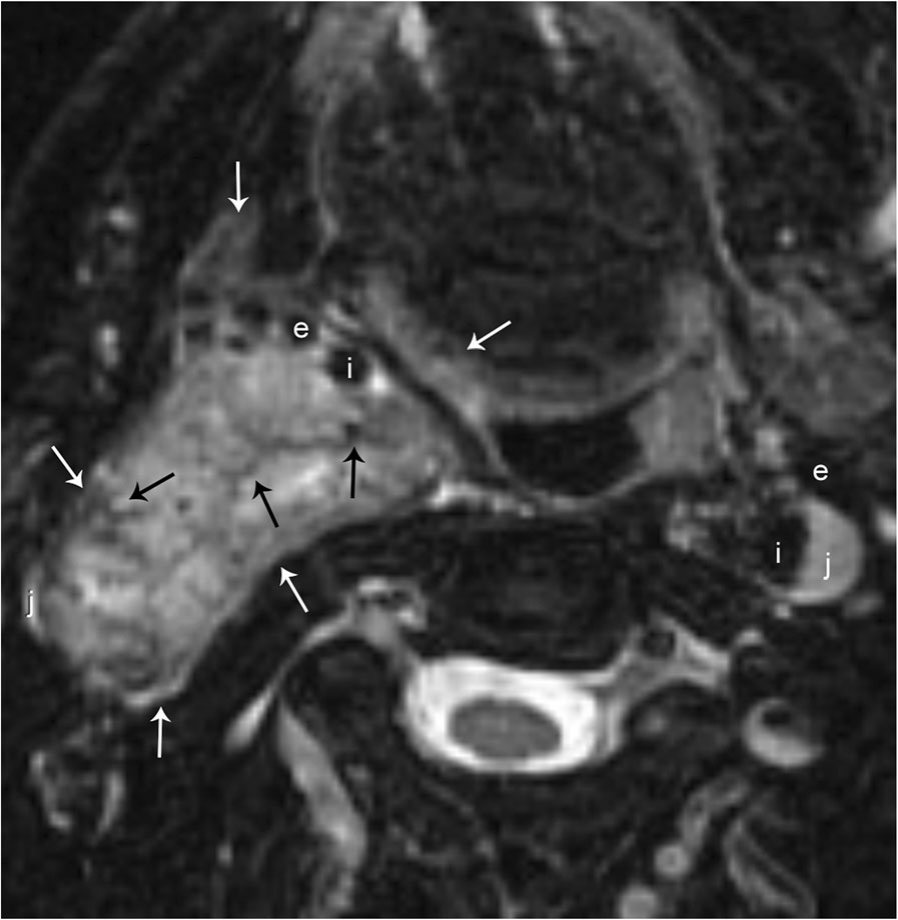
Glomus vagale. Axial T2-weighted MR image with fat suppression shows a well-circumscribed hyperintense mass (white arrows) with an internal “salt and pepper” appearance. This appearance is due to signal voids of tumor vessels (black arrows) and high signal areas due to slow flow and/or hemorrhage. Note the anterior displacement of the right internal (i) and external (e) carotid arteries, and lateral displacement of the right internal jugular vein (j). The vessels are labeled in their normal configuration on the left

**Fig. 5:**
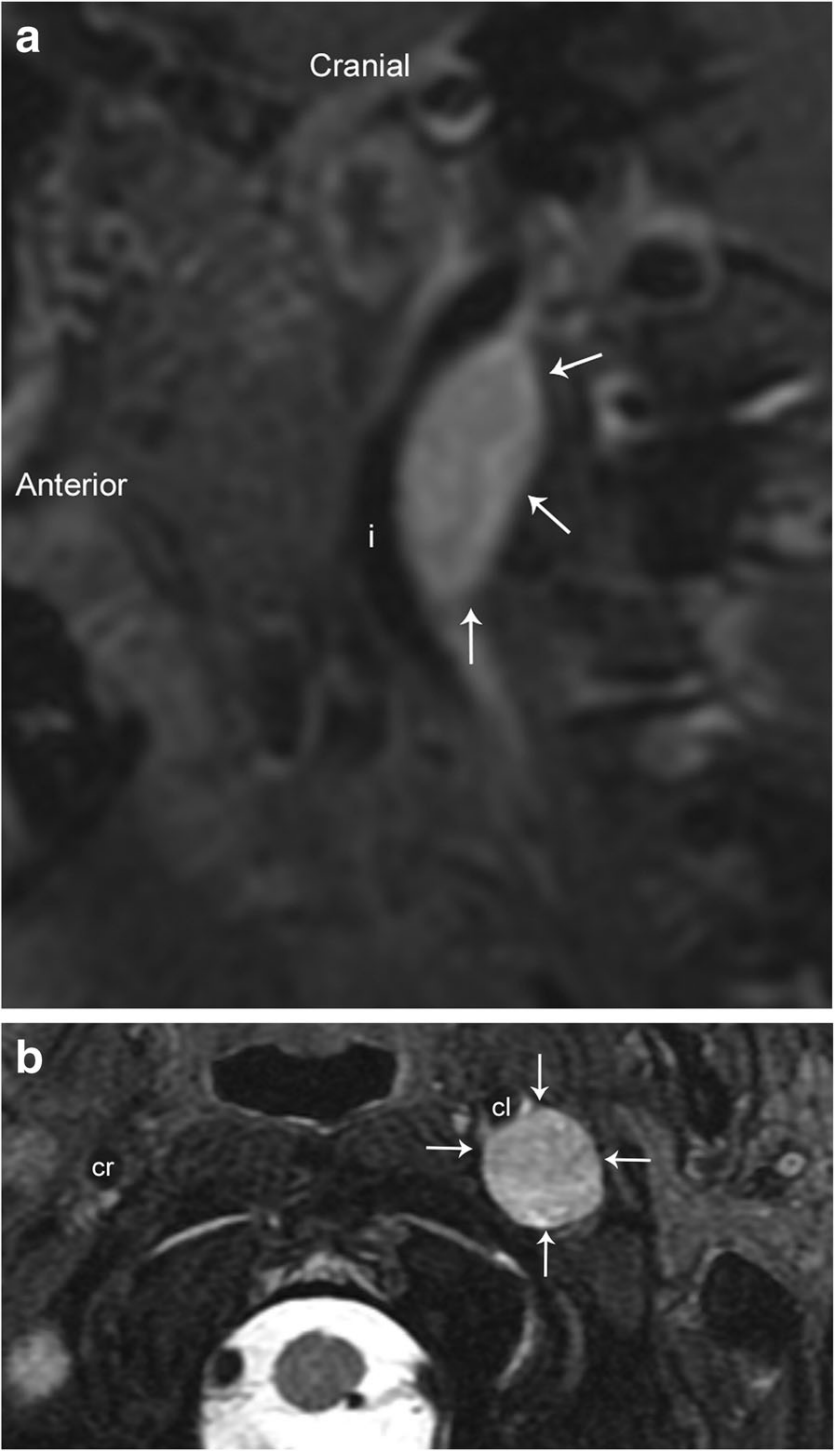
Glomus vagale. **a** Sagittal T1-weighted fat-saturated post-contrast image demonstrates anterior displacement of the left internal carotid artery (i) by a well-circumscribed and enhancing mass (arrows). **b** Axial T2-weighted fat-saturated image demonstrates the lesion to be hyperintense (arrows), causing anterior displacement of the left internal carotid artery (cl). The normal position of the right internal carotid artery (cr) is also seen

The vagus nerve is the longest cranial nerve, and although paragangliomas may arise anywhere along its tract, they are most commonly found at the ganglion nodosum (inferior ganglion) at the level of the C1 lateral mass [12]. These tumors have a heavy predilection for females in the fifth or sixth decades of life, however they are less common than the carotid body tumor or glomus jugulare [13]. Clinically, these present as asymptomatic masses posterior to the angle of the mandible; however, symptoms of vagal nerve dysfunction such as dysphagia, hoarseness, and vocal cord paralysis may develop late in the course. Although usually confined to the carotid space, these tumors can grow superiorly into the posterior fossa, entering via the jugular foramen, or inferiorly to the carotid bifurcation. As mentioned previously, the vagus nerve lies within the posterior aspect of the carotid space, and therefore, a large glomus vagale will anteriorly displace the carotid artery (Figs. 4 and 5).

### Nerve sheath tumors

**Fig. 6:**
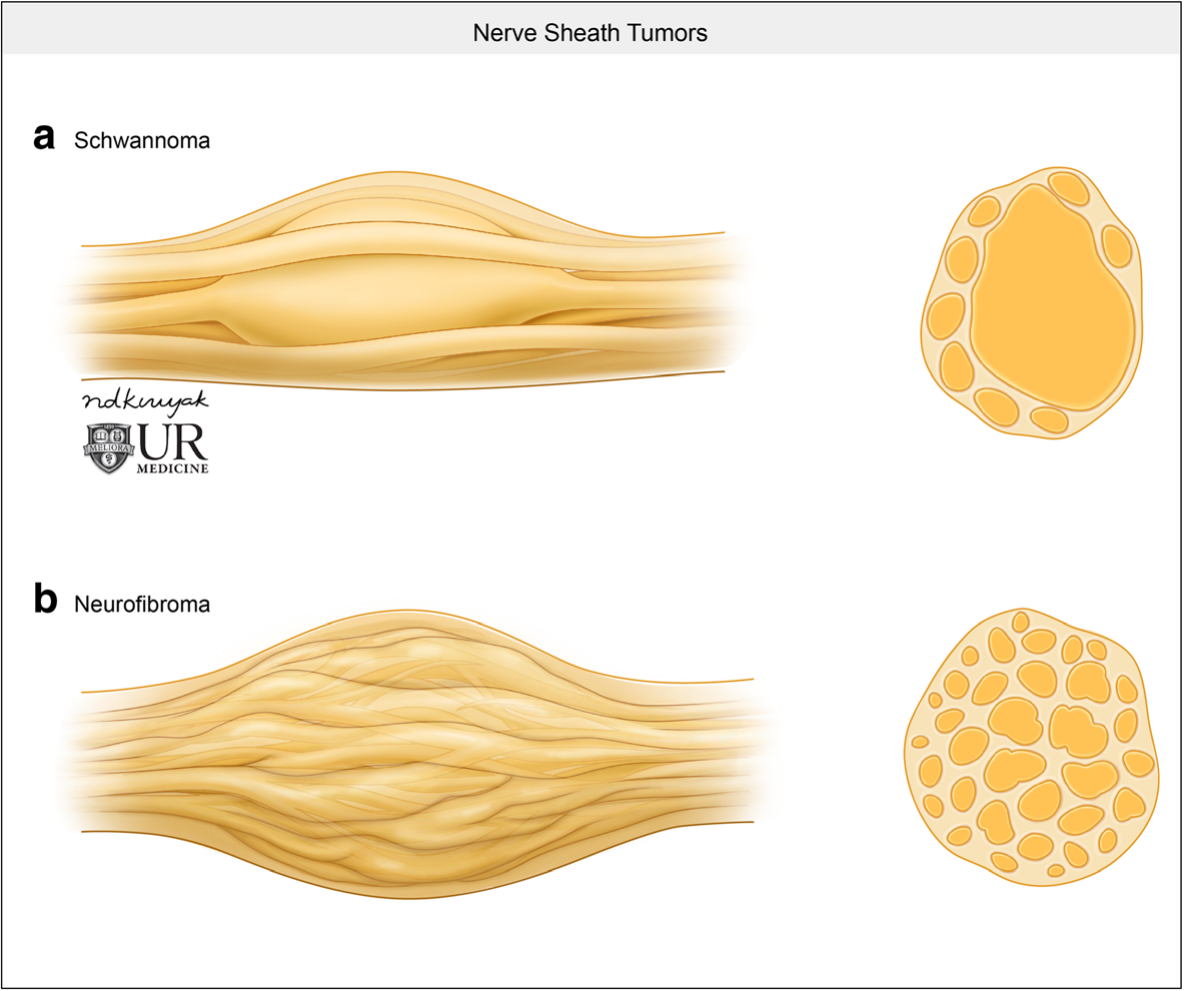
Nerve sheath tumors. **a** Illustration demonstrating the relatively eccentric configuration of a schwannoma relative to the affected nerve. **b** Conversely, neurofibromas tend to be fusiform, longitudinal, and interspersed in the nerve bundle

Primary neurogenic tumors that arise from nerve sheaths outside of the central nervous system are termed peripheral nerve sheath tumors. The overwhelming majority of these tumors are benign; however, malignant nerve sheath tumors can occur. Although major nerve trunks are most commonly affected, almost any peripheral nerve can be involved. Typically, peripheral nerve sheath tumors are categorized as either schwannoma or neurofibroma, both of which are associated with neurofibromatosis. Both categories of nerve sheath tumors have similar imaging findings; however, they can be differentiated by their configuration relative to the affected nerve (Fig. 6). On CT imaging, they tend to be hypoattenuating relative to muscle and enhance with contrast administration. On MRI, nerve sheath tumors present as low T1 signal and high T2 signal lesions which have avid contrast enhancement [14].

#### Neurofibroma

**Fig. 7:**
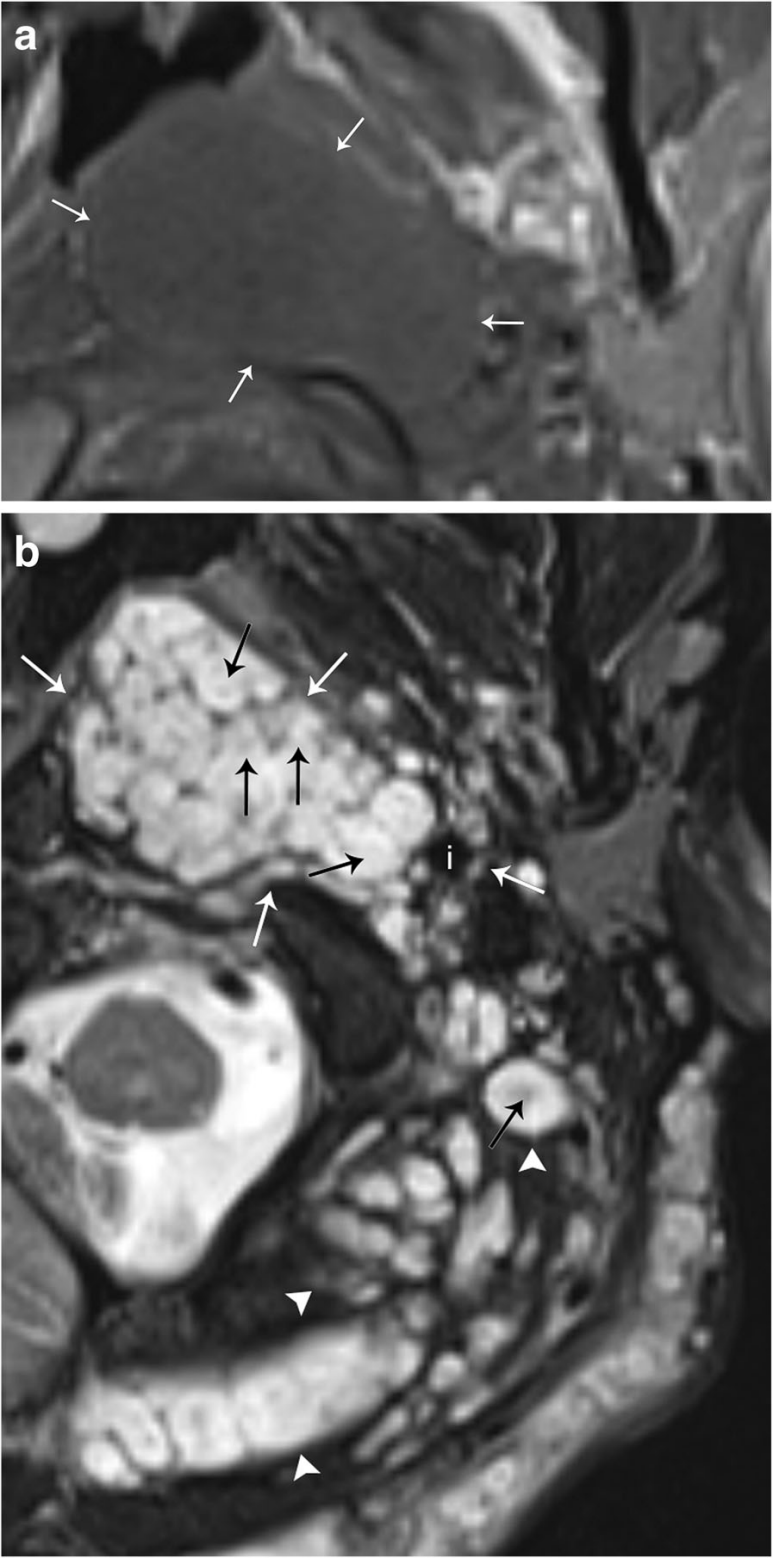
Plexiform neurofibroma. **a** Axial T1-weighted MR image demonstrates a mass that is iso−/hypointense to muscle (arrows). **b** Axial T2-weighted MR image demonstrates numerous hyperintense masses involving the left carotid space (white arrows). The left internal carotid artery (i) is displaced posterolaterally. Note the characteristic “fish-eye” or “target” appearance of this lesion (black arrows). This patient had neurofibromatosis, and extensive trans-spatial neurofibromas can be seen in other portions of the neck (arrowheads)

Neurofibromas are most frequently solitary lesions in young patients between 20 and 30 years of age. Ten percent of these neurofibromas are associated with neurofibromatosis and can be categorized as localized, plexiform, or diffuse. Diffuse neurofibromas are a subcutaneous lesion that do not present within the carotid space. Localized neurofibroma appear similar to solitary lesions but tend to be larger, multiple, and deeper in location. Due to a neurofibroma’s intimate association with the affected nerve, these tumors grow in a longitudinal and fusiform manner (Fig. 6). Tapered ends of the tumor, with an appearance of the parent nerve “entering and exiting,” the tumor can also be seen. A characteristic target sign or fish-eye appearance of the lesion referring to a central hypointense region has also been described [15]. Plexiform neurofibromas are pathognomic for neurofibromatosis type I (Fig. 7); these tumors are extensive and grow along a nerve and plexus and carry an increased risk of malignant transformation.

#### Schwannoma

**Fig. 8:**
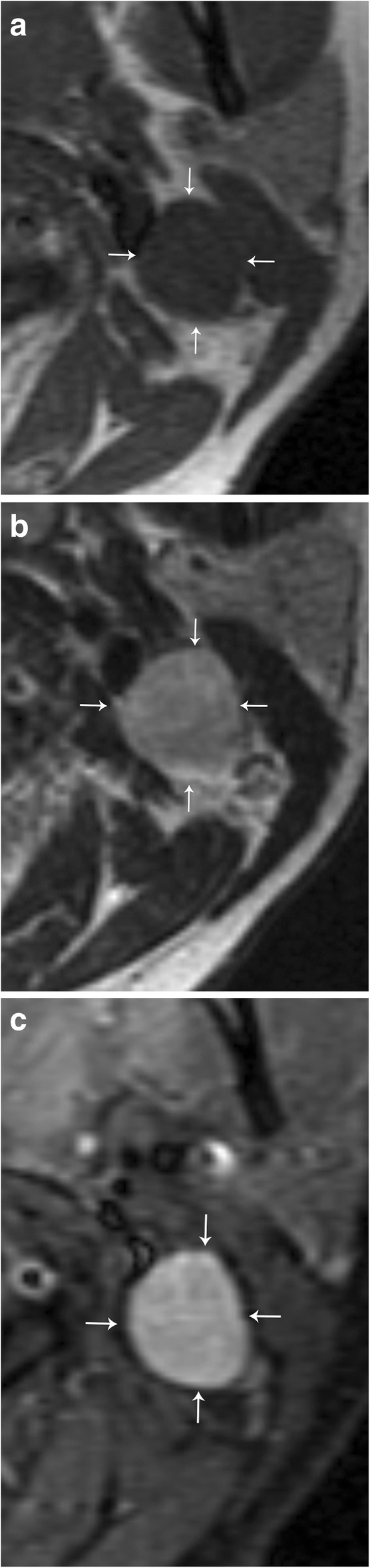
Schwannoma. **a** Axial T1 weighted MR image shows a well-circumscribed mass (arrows), which is low in signal. **b** T2-weighted MR image show that this mass is T2 hyperintense (arrows). **c** Post-contrast T1-weighted image shows avid and uniform enhancement of the mass (arrows)

**Fig. 9:**
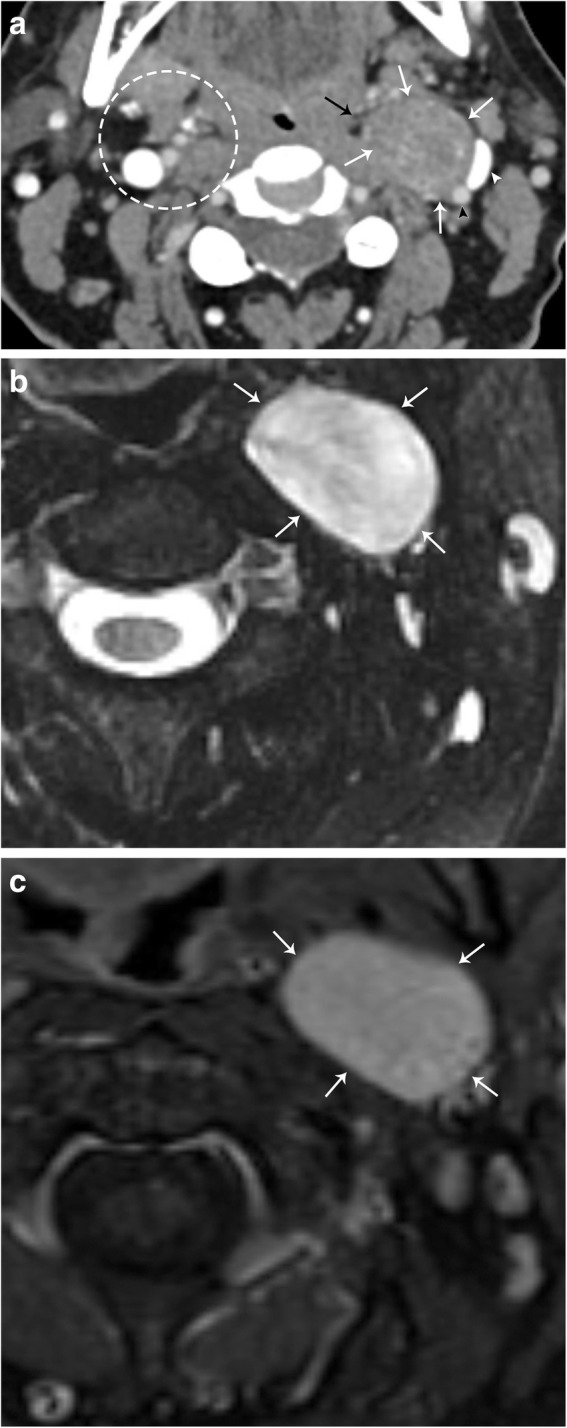
Sympathetic plexus schwannoma. **a** Axial CT image demonstrates a well-circumscribed mass (white arrows) that laterally displaces the left internal carotid artery (black arrowhead) and left internal jugular vein (white arrowhead). The left parapharyngeal fat is medially displaced (black arrow). Note the normal configuration of the right carotid space and parapharyngeal fat (dotted circle). **b** Axial T2 fat-suppressed MR image demonstrates homogenous hyperintensity of this mass (white arrows). **c** Axial T1 post-contrast fat-saturated MR image demonstrates homogenous enhancement of the lesion. Note the anteromedial location of the lesion relative to the carotid vessels, indicating sympathetic plexus origin

Schwannomas represent up to 5% of benign soft tissue neoplasms and typically present in patients 20–40 years of age. Most schwannomas are solitary; however, they are associated with neurofibromatosis, in which case they can be multifocal and plexiform as well. Schwannomas are also typically asymptomatic until late in the disease course, where neurologic symptoms associated with compression of the associated nerve may present. As mentioned previously, enhancement pattern of schwannoma can be similar to neurofibroma, and therefore, a salient differentiating imaging finding between the two is the eccentric position relative to the parent nerve in schwannoma (Figs. 6 and 8). Schwannomas are also more likely to be heterogeneous in appearance with areas of degeneration and cystic cavitation when large [15]. “Ancient” schwannoma, presenting with very advanced degeneration, calcification, and hyalinization, have also been described in the literature [14]. It is important to remember that in addition to schwannomas involving the cranial nerves, they may also be found within the sympathetic chain. These lesions will be located anteriorly or medially within the carotid space, displacing the carotid vessels laterally (Fig. 9).

### Lipoma

Lipomas are common, benign, well-circumscribed, and encapsulated soft masses comprised of mature adipocytes. Although these are most commonly found as subcutaneous nodules, they can be found anywhere in the body. These tumors demonstrate characteristic low (fat) attenuation on CT imaging and follow subcutaneous fat signal on all MRI sequences. Persistent areas of high T2 signal after fat saturation are a worrisome feature. When no suspicious features are present, MRI is 100% specific, and if suspicious features are present, MRI is 100% sensitive, and thus, MRI is the preferred imaging modality [16].

### Carotid sheath meningioma

**Fig. 10:**
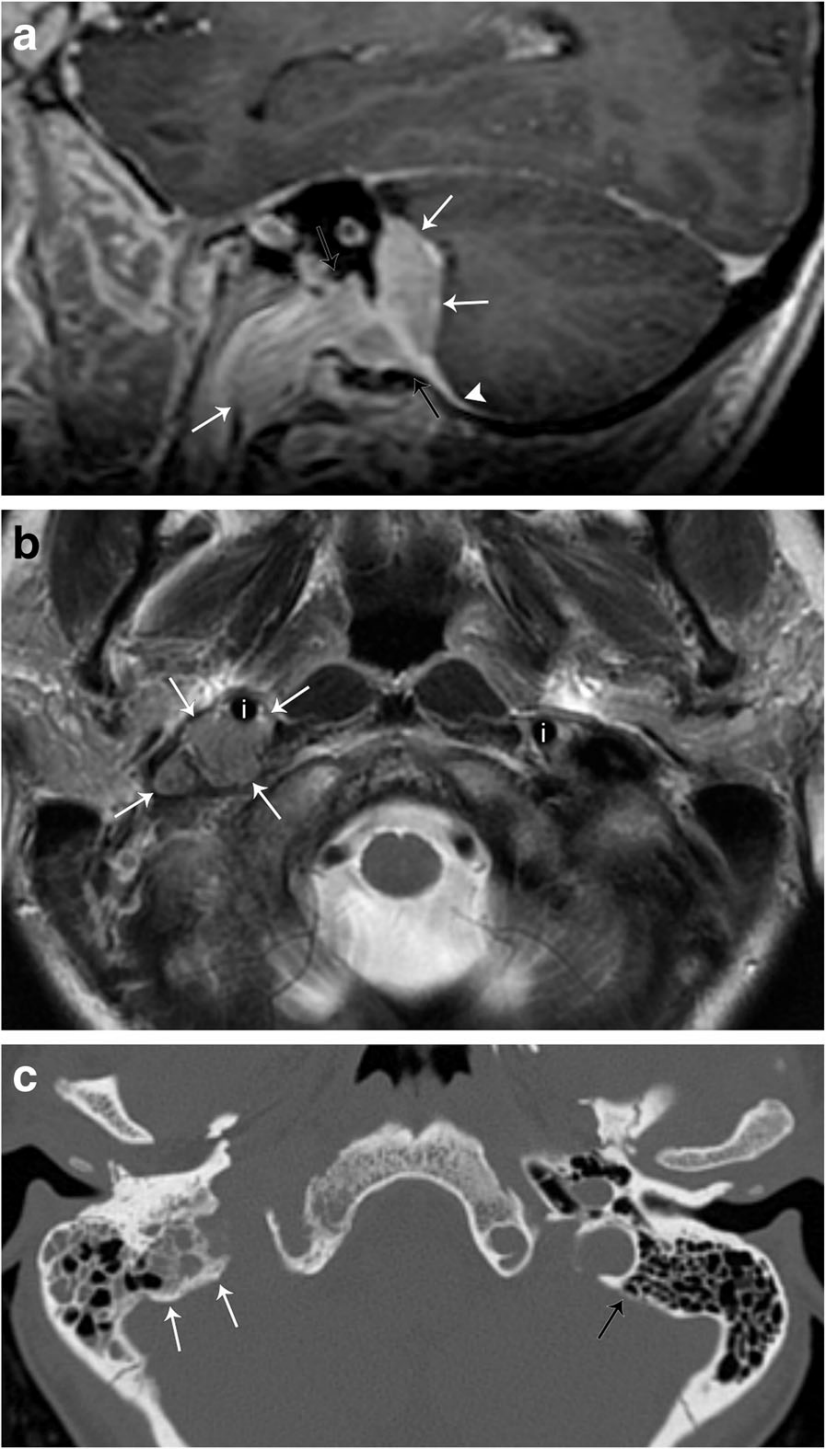
Meningioma. **a** Sagittal T1-weighted post-contrast MR image demonstrates an enhancing mass (white arrows) extending from the posterior fossa through the jugular foramen (black arrows) into the right carotid space. An enhancing dural tail is seen (arrowhead). **b** Axial T2-weighted MR image demonstrates the mass to be intermediate in signal (white arrows), anteriorly displacing the internal carotid artery (i). The normal position of the left internal carotid artery is also seen. **c** Axial non-contrast CT image demonstrates hyperostosis of the bone adjacent to the known lesion (white arrows). Note the thickness of the normal left mastoid bone cortex (black arrow)

Meningiomas are the most common extra-axial neoplasms of the central nervous system. Most meningiomas occur intracranially and are by definition closely associated with the dura. Tumors in the region of the skull base, in particular at the jugular foramen, can extend inferiorly and into the carotid space (Fig. 10). Primary extradural meningiomas are very rare, but have also been described, and are thought to originate from embryologic arachnoid rests. Meningiomas are typically mildly hyperattenuating on CT (60%), and up to 20% are associated with calcifications. The bone overlying meningioma is also often affected, most commonly with reactive hyperostosis. On MRI, meningiomas typically demonstrate iso- to hypointensity relative to gray matter on T1-weighted images and iso- to hyperintensity on T2-weighted images. A characteristic “dural tail” sign may be present, which confirms the tumors’ association with the dura. MRI can also detect sequelae of the mass effect such as venous sinus thrombosis and invasion, as well as parenchymal edema [17].

### Pathology of the carotid artery and internal jugular vein

The vascular components of the carotid space are also subject to a variety of pathologies. These may be congenital or acquired.

#### Carotid dissection

**Fig. 11:**
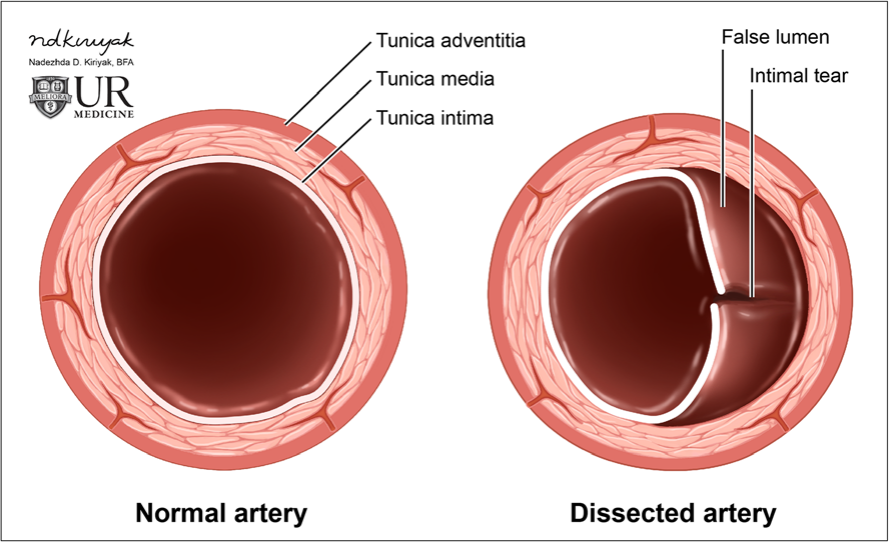
Arterial dissection. Illustration of the normal layers of an arterial vessel (left). Dissection occurs when a tear in the intimal layer allows blood to collect between the tunica intima and tunica media, creating a false lumen (right)

**Fig. 12:**
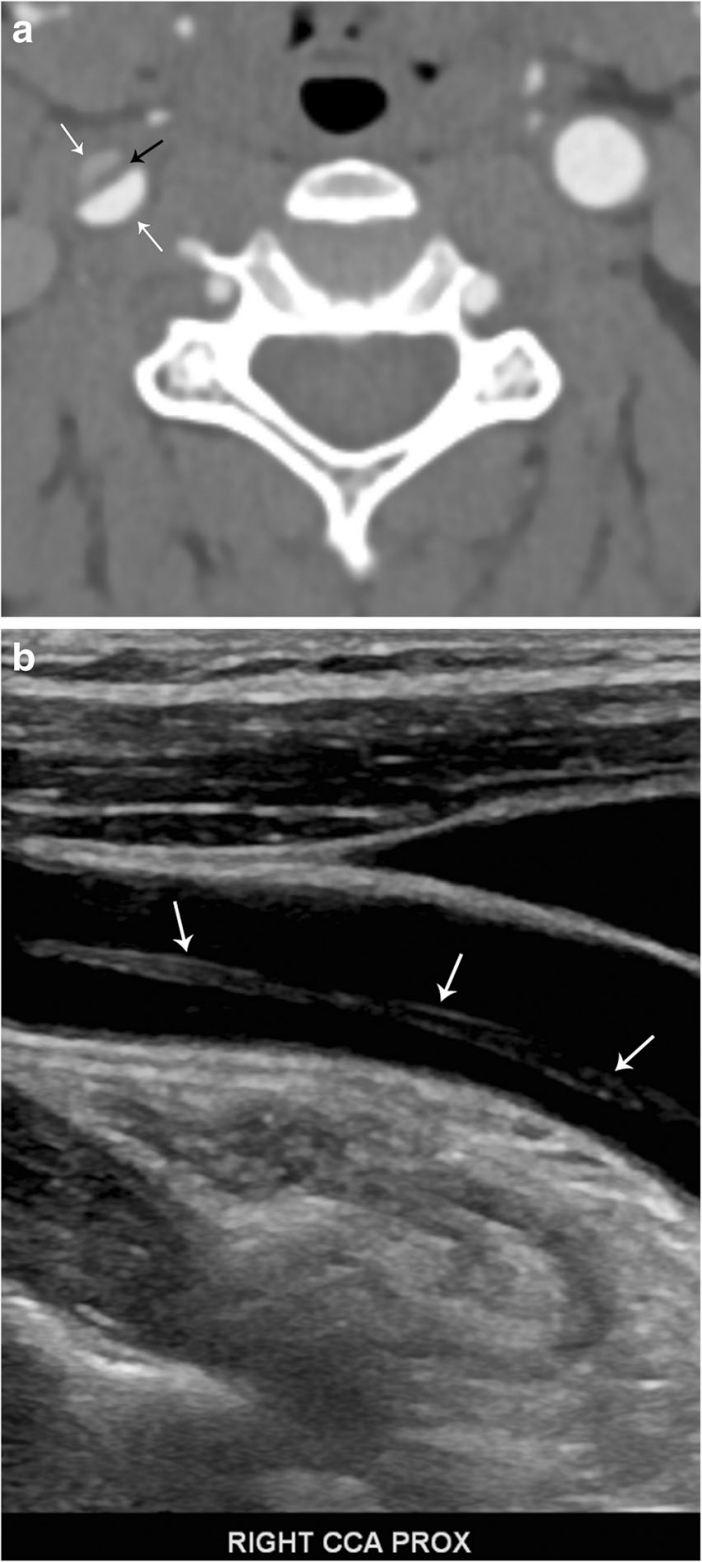
Carotid arterial dissection. **a** Axial contrast-enhanced CT image demonstrates an intimal flap (black arrow) and "double lumen" sign (white arrows) in the right common carotid artery. **b** Sagittal grayscale ultrasound image confirms the diagnosis, demonstrating the intimal flap (arrows)

Arterial dissection occurs when the innermost and least elastic layer of an arterial wall, the tunica intima, tears and allows the blood to enter, and form hematoma, in the tunica media (Figs. 11 and 12). An intimal tear can occur spontaneously, or may be related to trauma or iatrogenic causes. Regardless of the cause, it usually results in narrowing of the true vessel lumen. Carotid arterial dissection accounts for roughly 20% of causes of stroke in patients under 45 years of age. Typical symptoms include pain, ipsilateral Horner syndrome, and sequelae of brain ischemia [18]. It is important to remember the sympathetic fibers that carry innervation for a facial sweating run along the external carotid artery; thus, a dissection that involves only the internal carotid artery will only result in ptosis and meiosis, or a partial Horner’s syndrome [19]. A conventional angiogram is considered the gold standard for diagnosis but is invasive and only assesses luminal diameter and not arterial wall thickness or configuration. Color duplex ultrasound detects mural hematoma/thrombus as a thickened hypoechoic wall and can demonstrate the effects of luminal compromise dynamically. CT angiography offers a higher spatial resolution for evaluation of the intimal flap and vessel expansion than MRI, but is less sensitive for intracranial sequelae of ischemia and can be limited by artifact when in close proximity to the bone. MRI with T1 fat-suppressed sequences are highly sensitive for intramural blood, and post-contrast imaging can reliably characterize luminal narrowing [20].

#### Carotid pseudoaneurysm

**Fig. 13:**
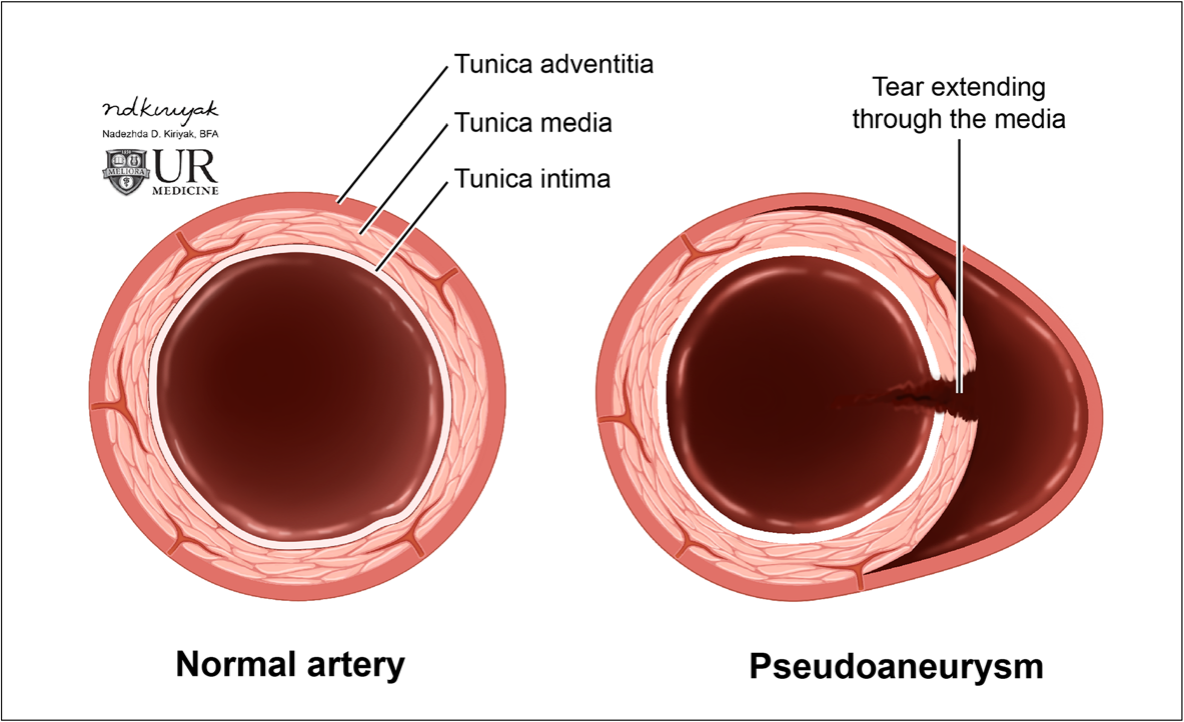
Arterial pseudoaneurysm. Illustration of the normal layers of an arterial vessel (left). Pseudoaneurysm occurs when a tear through the tunica intima and media layers results in blood only contained by the adventitial layer (right)

**Fig. 14:**
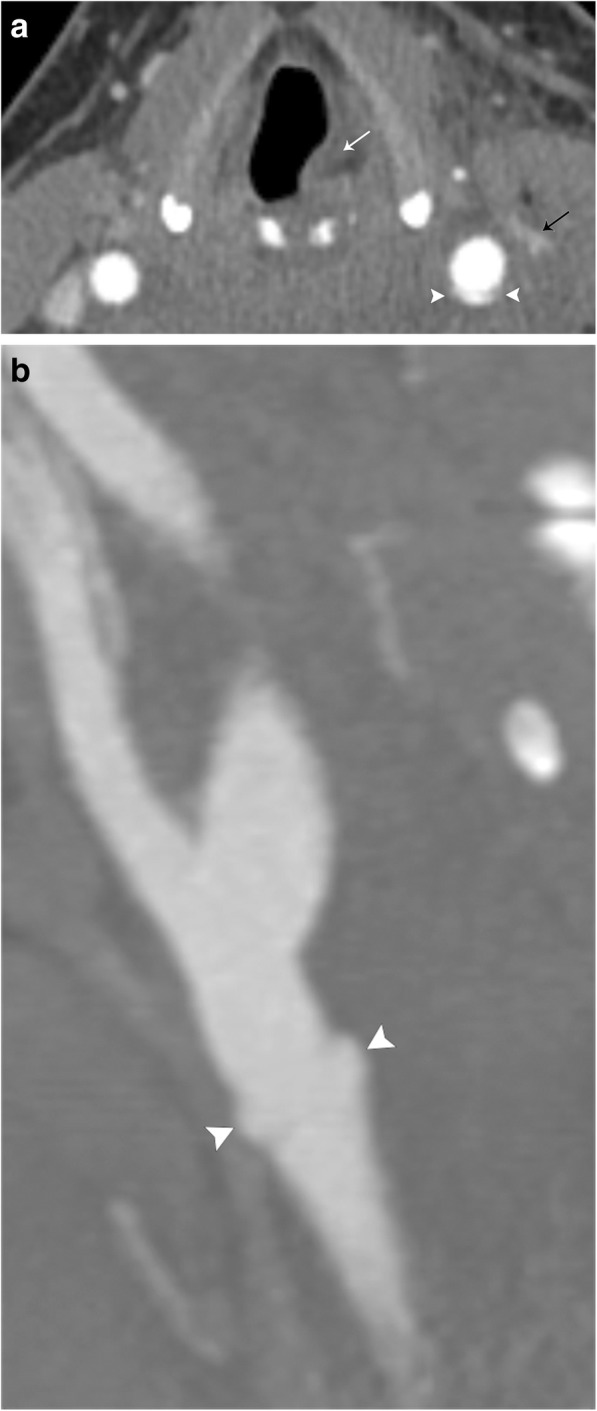
Traumatic carotid artery pseudoaneurysm. **a** Axial CT angiogram image demonstrates irregularity of the left common carotid artery (arrowheads) after stab wound to the neck. The left internal jugular vein (black arrow) is also injured. Note medialization (paralysis) of the left vocal fold (white arrow), due to concomitant vagus nerve injury. **b** Sagittal curved reformatted CT angiogram image better demonstrates the focal pseudoaneurysm (arrowheads) of the left common carotid artery, proximal to the bifurcation

**Fig. 15:**
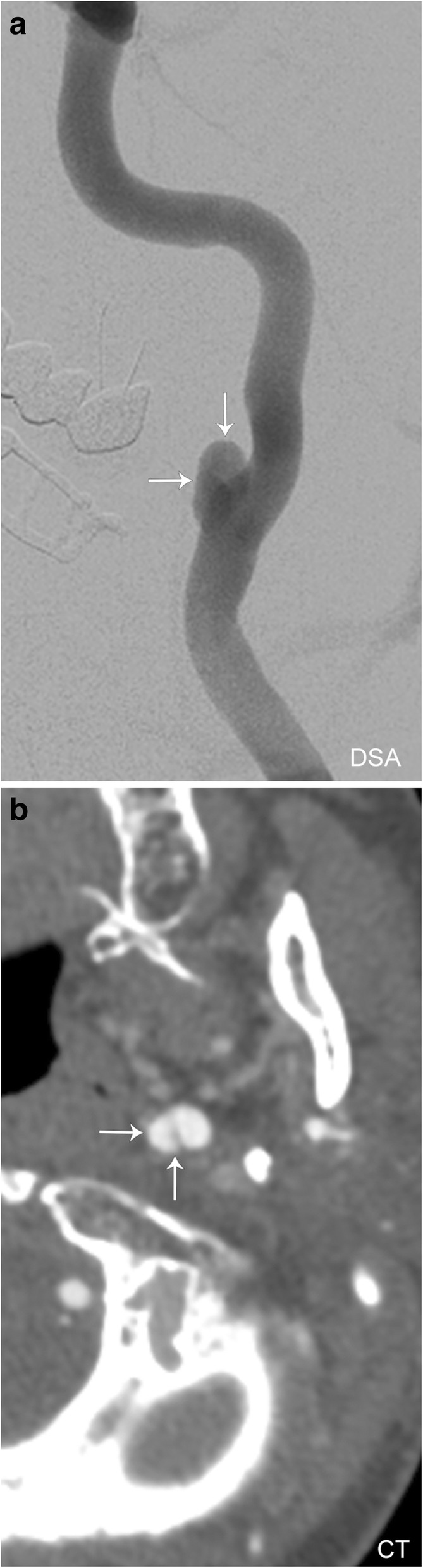
Iatrogenic pseudoaneurysm. **a** Conventional angiogram and **b** axial CT angiogram images demonstrating the appearance of a pseudoaneurysm (white arrows)

Pseudoaneurysm occurs when there is an injury of the tunica intima and media, resulting in a hematoma that is contained only by the thin outer adventitial layer of the vessel (Fig. 13). A pseudoaneurysm can form as a result of trauma (Fig. 14), dissection, vasculitis, infection, or be iatrogenic (Fig. 15). This entity can often be asymptomatic, and discovered incidentally; however, it carries a significant risk of rupture with high mortality rates. On ultrasound, a hypoechoic cystic structure can be demonstrated adjacent to the true vessel. Color duplex imaging can demonstrate a “to and fro” color flow and bidirectional Doppler waveform in the pseudoaneurysm neck. Within the sac itself, swirling blood can create the so-called “yin-yang” sign. CT imaging can demonstrate irregularity of a vessel wall and irregular outpouching. Hematoma with contrast extravasation seen on angiographic studies is compatible with rupture and active bleeding. When a pseudoaneurysm is large enough, it is important to note that the entire sac may not enhance, as the pseudoaneurysm can partially thrombose. MRI with T1-weighted fat-suppressed sequences allows for evaluation of intraluminal thrombus and of pseudoaneurysm sac size. Digital subtraction angiography remains the gold standard for evaluation of pseudoaneurysm and simultaneously offers a therapeutic potential [21].

#### Carotid thrombus

Carotid thrombus can occur as a result of direct trauma or in relation to severe carotid artery stenosis with atherosclerotic plaque. Patients with internal carotid artery thrombosis carry a 5 times higher rate of recurrent stroke than patients without thrombosis. Duplex sonography is capable of demonstrating intraluminal thrombus; however, CT angiography is more sensitive [22]. Magnetic resonance (MR) angiography can also demonstrate intraluminal filling defects, and concurrent MRI is important when looking for downstream sequelae of thrombus, such as infarct. In patients with occlusive thrombus, inflammatory changes about the carotid arteries including surrounding tissue edema, free fluid, and vessel expansion may also be visualized.

#### Fibromuscular dysplasia

**Fig. 16:**
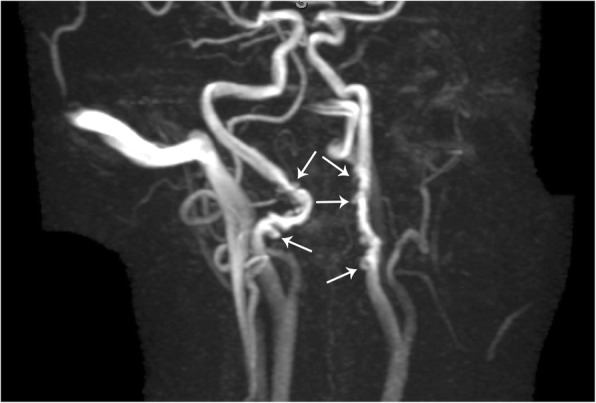
Fibromuscular dysplasia. MR angiography image of the bilateral internal carotid arteries demonstrates a "string-of-beads" appearance (arrows)

Fibromuscular dysplasia of the carotid vessels is an uncommon and often asymptomatic pathology and therefore often an incidental finding. However, symptomatic patients can present with cerebral infarction, transient ischemia, or subarachnoid hemorrhage. Although fibromuscular dysplasia can occur in any of the three arterial wall layers, the involvement of the tunica media is most common, representing over 90% of cases. The arterial wall becomes dysplastic due to fibrous thickening and destruction of the elastic lamina, often leading to multifocal stenosis and areas of luminal expansion (Fig. 16). It is important to note that the mural dilation is often greater than the normal vessel diameter, thus helping distinguish fibromuscular dysplasia from atherosclerotic disease. This yields the characteristic “string-of-beads” appearance of the dysplastic vessel, a sign that is seen in over 80–90% of cases. In severe cases, fibrous dysplasia can be complicated by arterial dissection, saccular aneurysm formation, or arteriovenous fistula formation. CT angiography and MRI angiography are both utilized regularly; however, CTA is preferred due to being readily available, fast scan time, higher spatial resolution, and decreased potential for artifact [23].

#### Carotidynia

**Fig. 17:**
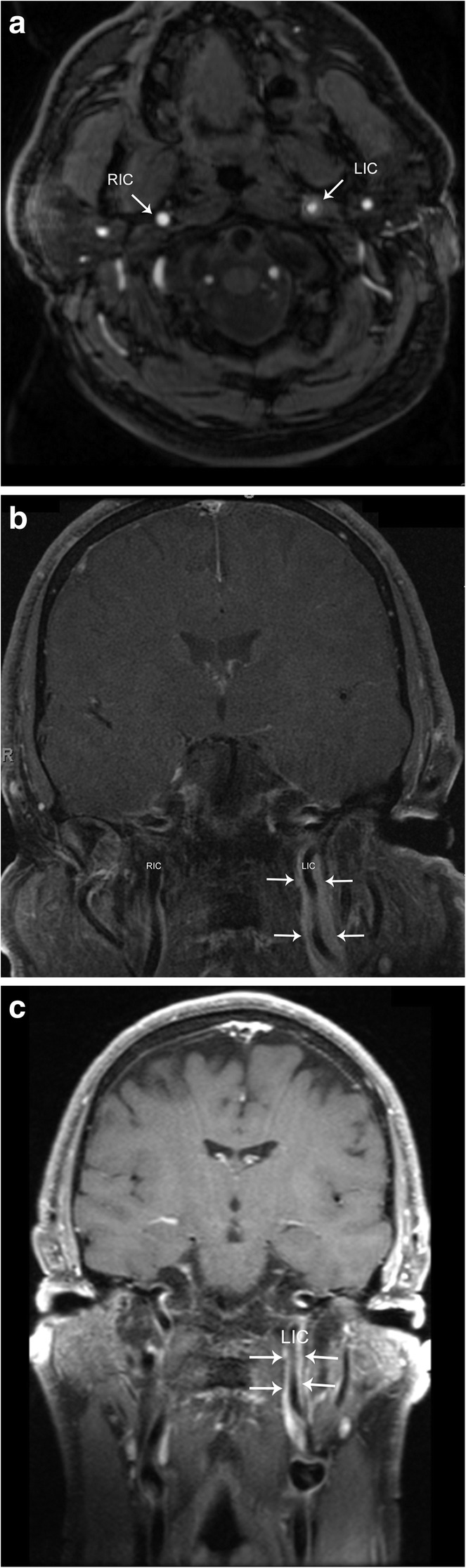
Carotidynia. **a** Axial time of flight image demonstrates a rind of soft tissue surrounding the left internal carotid artery (LIC). Note the normal appearance of the right internal carotid artery (RIC). **b** Coronal post-contrast T1-weighted image shows avid enhancement of the rind of soft tissue (arrows) surrounding the left internal carotid artery (LIC). Note the normal appearance of the right internal carotid artery (RIC). **c** Follow-up approximately 4 months later, coronal post-contrast T1-weighted fat-saturated image shows decreased thickness of the rind of soft tissue (arrows)

Also known as Fay syndrome, carotidynia is a rare entity characterized by unilateral pain in the region of the carotid bifurcation. Pain tends to be pulsating and continuous in sensation, but is a self-limiting process, and often spontaneously resolves in 1–2 weeks. Laboratory workup is negative for immunological markers, and patients are often treated with NSAIDs. Although controversial, the etiology of carotidynia is stipulated to be related to an idiopathic inflammatory process of the carotid bifurcation and, therefore, has recently been termed transient perivascular inflammation of the carotid artery syndrome (TIPIC). Ultrasound, CT, and MRI of the carotid arteries demonstrate fusiform transmural thickening without luminal compromise. Doppler ultrasound demonstrates normal flow velocities without evidence of turbulent flow, as opposed to other pathologies that may cause narrowing of the lumen (such as severe atherosclerotic disease). CT and MRI fail to demonstrate signs of vascular inflammation outside of the carotid vessels. MRI demonstrates hypointensity of the vessel wall on fat-saturated T1-weighted imaging, precluding the possibility of a mural hematoma. Post-contrast MR images demonstrate intense enhancement of the carotid bifurcation, supporting the proposed inflammatory etiology (Fig. 17). Follow-up imaging after resolution of symptoms usually demonstrates a normal appearance of the carotid bifurcation [24].

#### Internal jugular vein thrombosis

**Fig. 18:**
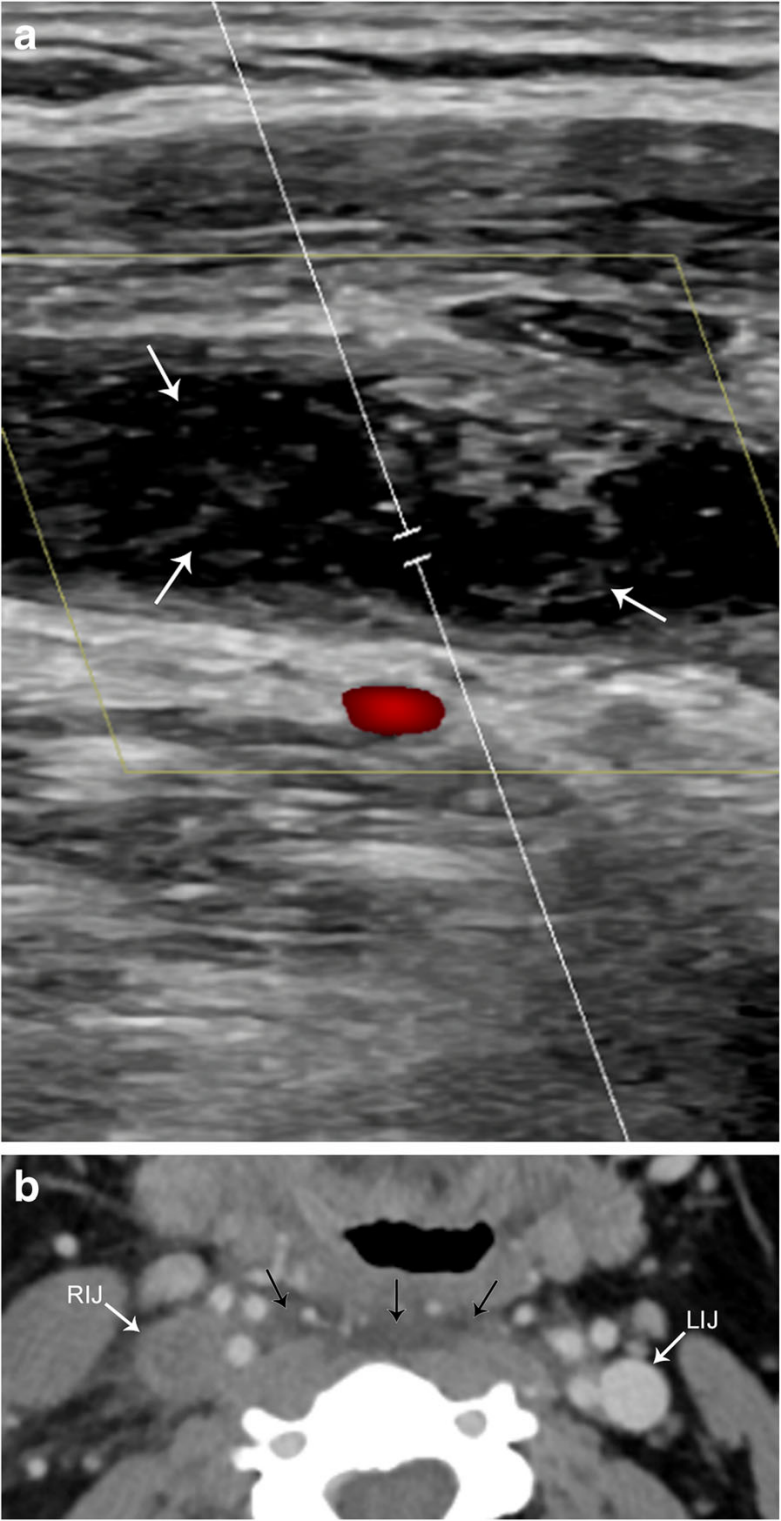
Acute jugular vein thrombosis. **a** Sagittal color flow Doppler ultrasound image demonstrates an occlusive hypoechoic thrombus (arrows) within the jugular vein. There is an absence of color fill within the vessel. **b** Axial contrast-enhanced CT image shows an expanded and non-enhancing right internal jugular vein (RIJ). Compare to the normal left internal jugular vein (LIJ). Note the retropharyngeal fluid (arrows), a common finding with acute jugular venous thrombosis

The paired internal jugular veins drain the majority of blood from the cranium, serving as cervical extensions of the dural venous sinuses. Thrombosis of the internal jugular veins can occur for many reasons, including extrinsic compression, chronic indwelling catheter, recent surgery, head and neck infections (Lemierre syndrome), malignancy, hypercoagulability syndromes, an extension of dural sinus thrombosis, and spontaneous thrombosis. Venous duplex ultrasonography is the diagnostic test of choice and demonstrates a dilated and incompressible vein; the intraluminal thrombus is often visualized as low-level echogenicity (Fig. 18). There is a limited evaluation of the vessels behind the clavicle and sternum, but Doppler ultrasound can demonstrate abnormal, monophasic waveforms due to a more proximal occlusive thrombus, and no significant flow change with Valsalva maneuver. CT findings of IJ thrombus include vessel expansion, surrounding inflammatory change and fluid/edema within the retropharyngeal space (Fig. 18) [25]. Contrast-enhanced CT can also help delineate the intraluminal thrombus, and enhancement of the vessel wall can be seen due to contrast uptake by the vasa vasorum. MRI is not used routinely for primary diagnosis of vein thrombosis due to expense and inconvenience in the acute setting; however, similar findings of vessel expansion, surrounding inflammatory change and T1 hyperintense intraluminal filling defect can be visualized.

### Pathology of the deep cervical lymph node chain

The cervical lymph nodes have well-defined regional classification criteria and myriad pathology; the complete discussion of which is beyond the scope of this paper. In general, lymph nodes are classified as abnormal when they measure greater than 1 cm in the short axis or demonstrate other size independent indicators of pathology such as loss of the fatty hilum, rounded shape, and focal necrosis or cystic change.

### Infectious

**Fig. 19:**
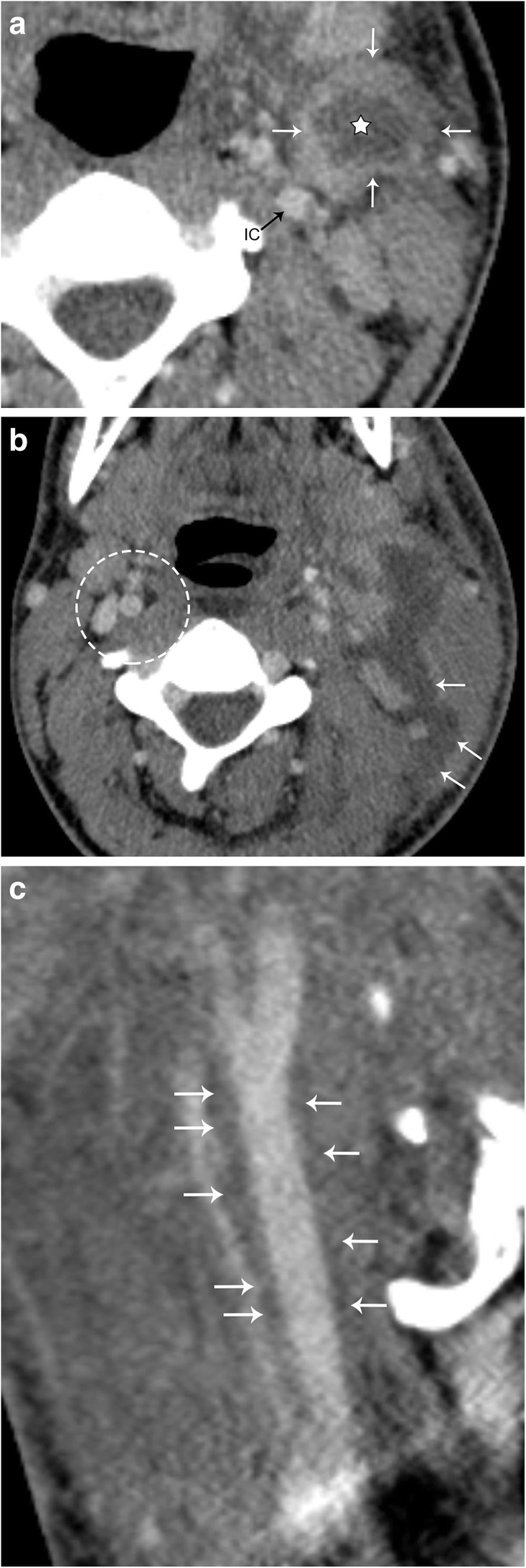
Suppurative lymph node. Patient presenting with fever, sore throat, cough, and a palpable left neck mass. **a** Axial contrast-enhanced CT image demonstrates an enhancing left deep cervical chain lymph node (white arrows), with a hypoattenuating center (star). The left internal carotid artery (IC) is medially displaced. **b** Axial contrast-enhanced CT image 3 days later demonstrates rupture (arrows) of the suppurative node. Note the preserved fat planes in the normal right carotid space (dotted circle). **c** Sagittal CT image demonstrates fluid tracking along the left carotid space from the lymph node rupture (arrows)

Infections in the head and neck can result in cervical adenitis, or inflammatory change of the deep cervical lymph nodes. The most common cause of cervical adenitis is a viral infection of the upper respiratory tract; however, there is a broad differential including bacterial infection, tuberculosis, and HIV. Affected lymph nodes are generally enlarged (greater than 1 cm in the short axis). In advanced infections, lymph nodes can undergo liquefactive necrosis, and are then referred to as suppurative lymph nodes. If left untreated, the suppurative lymph node may rupture, spilling into the deep spaces of the neck (Fig. 19). The most common causative agents for suppurative adenitis are *Staphylococcus aureus* and group A streptococcus bacterium [26]. CT scan with contrast is the exam of choice to evaluate these patients, since it is quick and readily available in the emergency setting.

#### Neoplastic

**Fig. 20:**
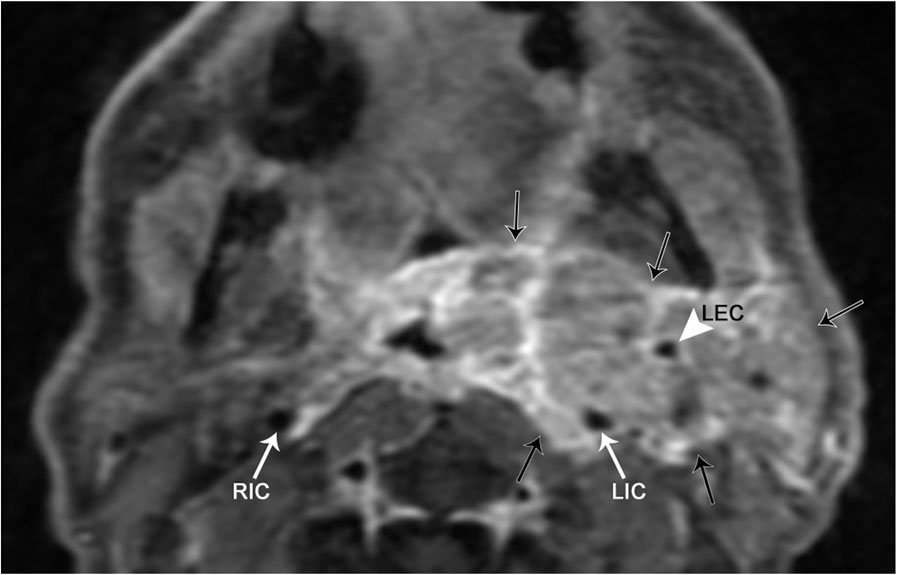
Lymphoma. Axial T1 post-contrast fat-suppressed image demonstrates a conglomerate of heterogeneously enhancing masses that completely encase the left internal (LIC) and external (LEC) carotid arteries. Note that the lumen of the LIC is similar in caliber to the right internal carotid artery (RIC)

Hodgkin lymphoma (HL) and non-Hodgkin lymphoma (NHL) commonly involve the head and neck. HL and NHL cannot be reliably distinguished via imaging, however should be considered in patients with B symptoms (fever, unintentional weight loss, or night sweats). Lymphoma typically presents as painless or growing lymphadenopathy [27]. On CT imaging, lymphomatous involvement can have varying degrees of enhancement, and involvement can be extensive. A conglomeration of lymph nodes may insinuate within the deep spaces of the neck, encasing important structures. It is important to remember that lymphoma does not typically cause luminal narrowing of arterial vasculature, a feature which may help distinguish the disease from others (Fig. 20). Although CT is the gold standard for the initial evaluation and staging of lymphoma, it only provides structural information. Positron emission tomography (PET) has an increasing role in lymphoma evaluation as it adds functional information. PET is the key to assessing disease response to therapy, as well as finding residual or recurrent disease after treatment.

**Fig. 21:**
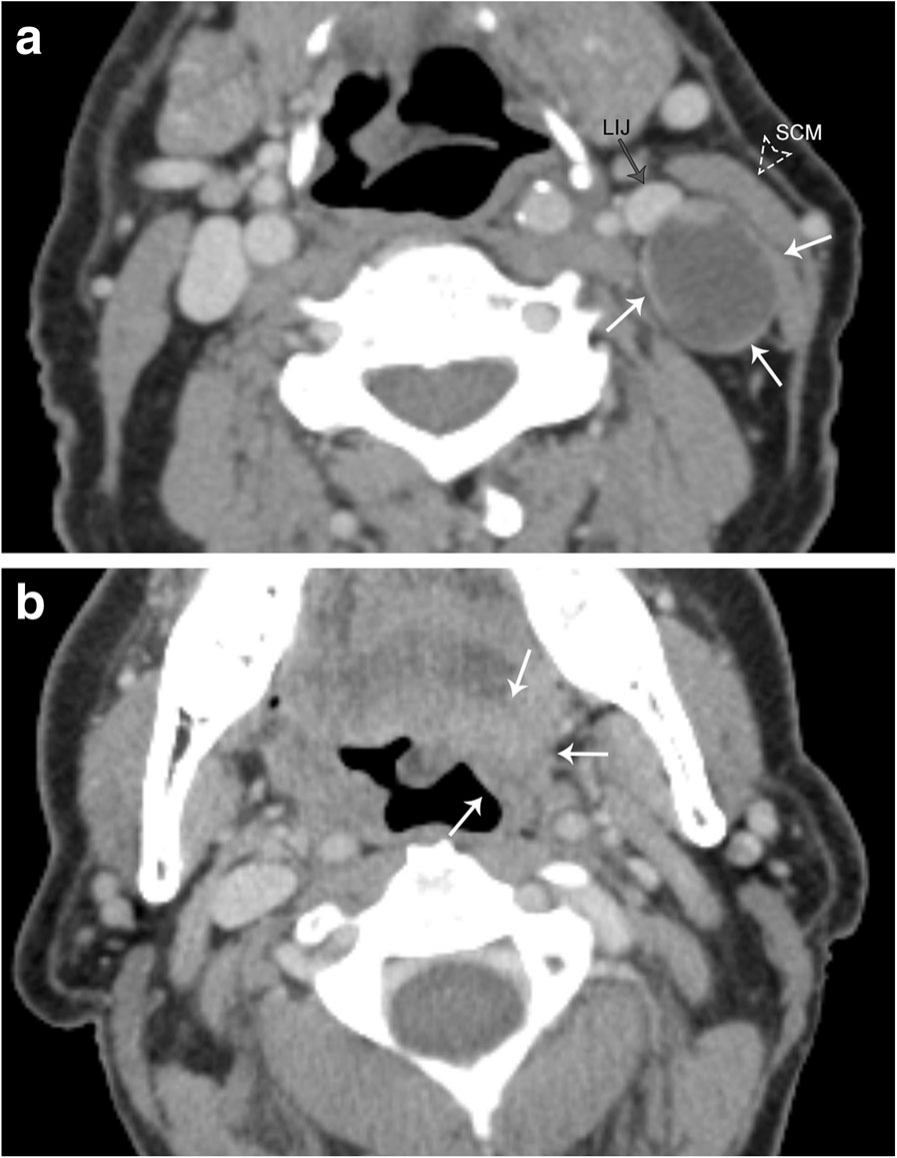
Cystic metastatic lymph node. Patient presenting with a palpable left neck mass, no other symptoms. **a** Axial contrast-enhanced CT image demonstrates a well-circumscribed, low attenuation lymph node in the left carotid space (white arrows); note some areas of peripheral nodularity. This node causes mass effect with anterior displacement of the left internal jugular vein (LIJ), and lateral displacement of the sternocleidomastoid muscle (SCM). **b** Axial contrast-enhanced CT image at a higher level in the same patient demonstrates an ill-defined mass centered at the left tongue base (arrows). Biopsy of this lesion demonstrated squamous cell carcinoma

Metastatic disease is also a common cause of adenopathy in the deep cervical chain. Although squamous cell carcinoma is the most common (representing 90% of head and neck cancers) [28], many other malignancies including EBV-related nasopharyngeal carcinoma, thyroid carcinoma, breast carcinoma, melanoma, and even lung carcinoma are also seen to metastasize to the neck lymph nodes [29]. Squamous cell carcinoma and papillary thyroid carcinoma are well known to demonstrate cystic nodal metastasis (Fig. 21) [30].

**Fig. 22:**
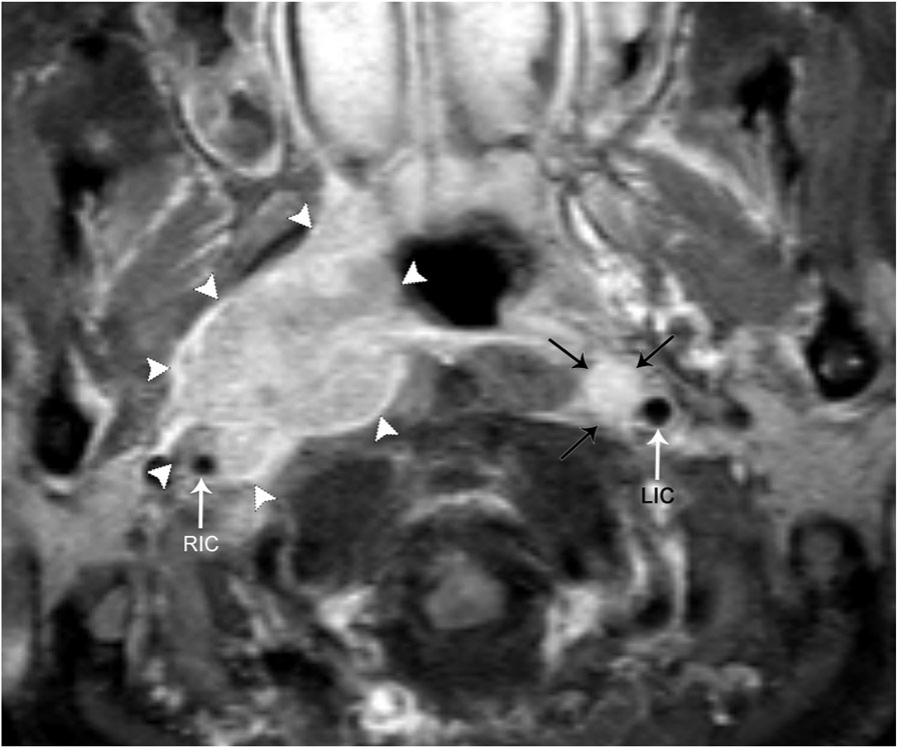
Direct spread of non-HPV-associated squamous cell carcinoma into the carotid space. Axial contrast-enhanced, fat-suppressed T1 image demonstrates a large, avidly enhancing and infiltrating mass that invades the right carotid space (white arrowheads). The right carotid artery (RIC) is encased, narrowed, and laterally displaced when compared to the left internal carotid artery (LIC). Encasement of the carotid artery upstages the disease to T4b. There is also a left retropharyngeal lymph node (black arrows), just medial to the LIC

It is important to remember that primary malignancy of the head and neck has the potential to directly invade the carotid space, which ultimately has staging and treatment implications. For example, in the setting of oropharyngeal non-HPV associated squamous cell carcinoma, encasement of the carotid artery upstages the lesion to T4b disease using the American Joint Committee on Cancer (AJCC) 8th edition criteria (Fig. 22) [31].

#### Mimics

**Fig. 23:**
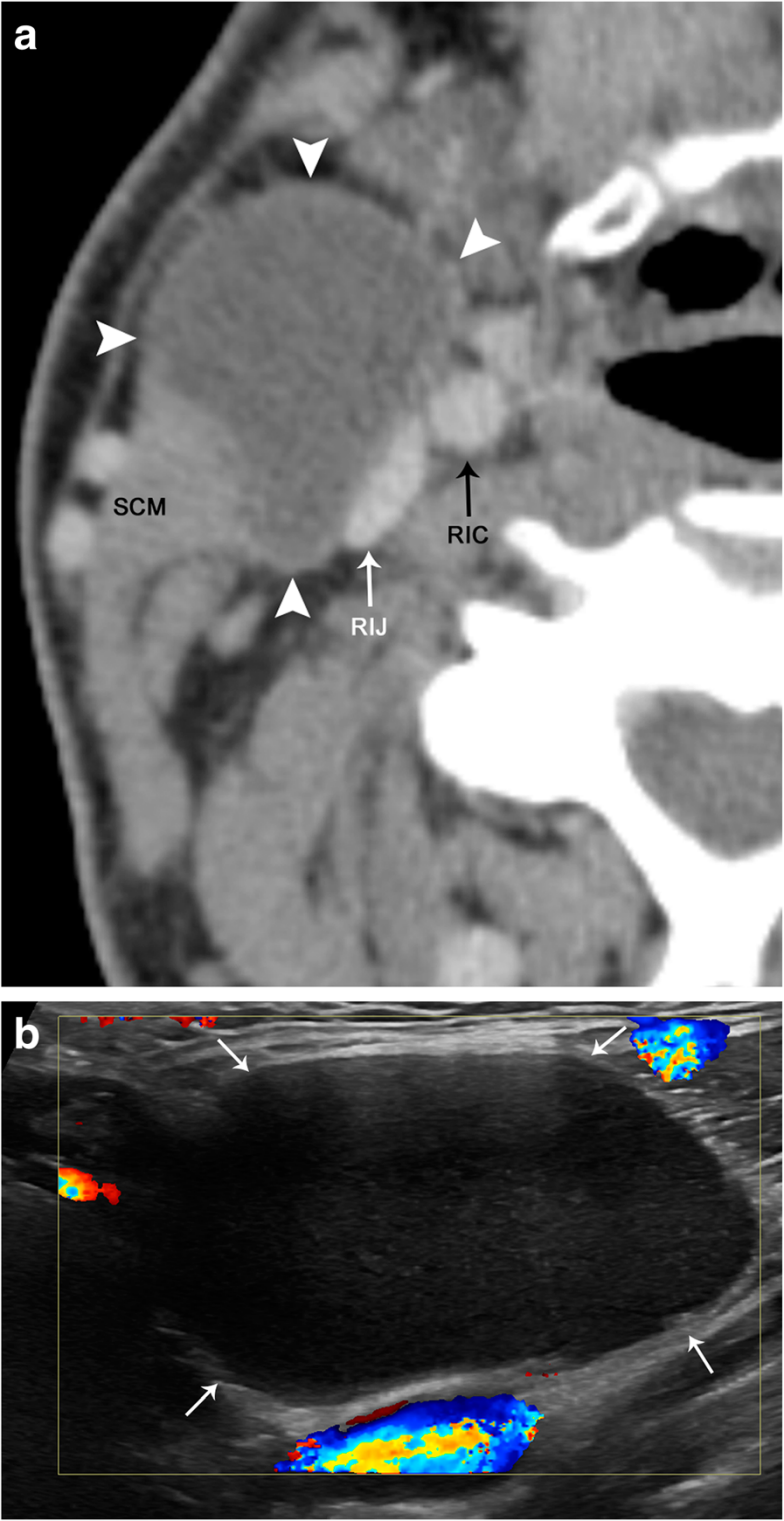
Second branchial cleft cyst. **a** Axial contrast-enhanced CT image demonstrates a well-circumscribed and hypoattenuating lesion (arrowheads) along the anterior margin of the right sternocleidomastoid muscle (SCM) and anterolateral to the right carotid space, internal carotid artery (RIC), and internal jugular vein (RIJ). **b** Color Doppler ultrasound image demonstrates that this lesion (arrows) is hypoechoic with low-level echoes. There is no internal vascularity or peripheral hyperemia

Incomplete obliteration of a branchial tract during development can result in a branchial cleft cyst (BCC). The second branchial cleft yields 95% of all branchial lesions. It is important not to confuse a developmental anomaly such as a BCC for a cystic lymph node, as the former is a completely benign entity. These lesions are usually well-defined, thin-walled cystic structures. A second BCC typically occurs lateral to the carotid space, along with the anterior margin of the sternocleidomastoid muscle, and can extend deep to the muscle (Fig. 23). Occasionally, these cysts may demonstrate a “beaked” appearance, when they invaginate between the internal and external carotid arteries. They can occasionally be complex and demonstrate low-level echoes on ultrasound, as well as internal septa. These lesions do not typically appear hyperemic on ultrasound or enhance on CT and MR; however, if they are infected or inflamed, thickening of the wall with intense enhancement and surrounding fat stranding can also be seen [32].

## Conclusion

Clearly, the carotid space has a complex anatomy confined to a small space. Knowledge of the anatomy within this space allows for the radiologist to correctly identify pathology. If a lesion involving the carotid space is suspected, one must interrogate from the level of the skull base at the jugular foramen inferiorly to the mediastinum. Pathology involving or occurring along cranial nerves 9 through 12, the carotid artery, and internal jugular vein, as well as deep cervical lymph node chain, must all be considered.
